# Data-driven Thyroglobulin Cutoffs for Low- and Intermediate-risk Thyroid Cancer Follow-up: ITCO Real-world Analysis

**DOI:** 10.1210/clinem/dgae559

**Published:** 2024-08-16

**Authors:** Giorgio Grani, Silvia D’Elia, Efisio Puxeddu, Silvia Morelli, Emanuela Arvat, Alice Nervo, Giovanna Spiazzi, Nicoletta Rolli, Maria Chiara Zatelli, Maria Rosaria Ambrosio, Graziano Ceresini, Michela Marina, Chiara Mele, Gianluca Aimaretti, Maria Giulia Santaguida, Camilla Virili, Anna Crescenzi, Andrea Palermo, Ruth Rossetto Giaccherino, Letizia Meomartino, Maria Grazia Castagna, Fabio Maino, Matteo Trevisan, Simone De Leo, Maria Grazia Chiofalo, Luciano Pezzullo, Clotilde Sparano, Luisa Petrone, Giulia Di Dalmazi, Giorgio Napolitano, Dario Tumino, Umberto Crocetti, Francesco Bertagna, Maurilio Deandrea, Alessandro Antonelli, Caterina Mian, Antonella Carbone, Salvatore Monti, Tommaso Porcelli, Giulia Brigante, Daniele Barbaro, Marco Alfò, Umberto Ferraro Petrillo, Sebastiano Filetti, Cosimo Durante

**Affiliations:** Department of Translational and Precision Medicine, Sapienza University of Rome, 00161 Rome, Italy; Department of Statistical Sciences, Sapienza University of Rome, 00185 Rome, Italy; Department of Medicine and Surgery, University of Perugia, 06129 Perugia, Italy; Department of Medicine and Surgery, University of Perugia, 06129 Perugia, Italy; Oncological Endocrinology Unit, Department of Medical Sciences, Città della Salute e della Scienza Hospital, University of Turin, 10126 Turin, Italy; Oncological Endocrinology Unit, Department of Medical Sciences, Città della Salute e della Scienza Hospital, University of Turin, 10126 Turin, Italy; Endocrinology and Diabetology Unit, Department of Medicine, Azienda Ospedaliera-Universitaria di Verona, 37126 Verona, Italy; Endocrinology and Diabetology Unit, Department of Medicine, Azienda Ospedaliera-Universitaria di Verona, 37126 Verona, Italy; Section of Endocrinology, Geriatrics and Internal Medicine, Department of Medical Sciences, University of Ferrara, 44121 Ferrara, Italy; Section of Endocrinology, Geriatrics and Internal Medicine, Department of Medical Sciences, University of Ferrara, 44121 Ferrara, Italy; Unit of Internal Medicine and Oncological Endocrinology, University Hospital of Parma, 43126 Parma, Italy; Unit of Internal Medicine and Oncological Endocrinology, University Hospital of Parma, 43126 Parma, Italy; Endocrinology, Department of Translational Medicine, University of Piemonte Orientale, 28100 Novara, Italy; Endocrinology, Department of Translational Medicine, University of Piemonte Orientale, 28100 Novara, Italy; Endocrinology Unit, Department of Medico-Surgical Sciences and Biotechnologies, Sapienza University of Rome, 04100 Latina, Italy; Endocrinology Unit, Department of Medico-Surgical Sciences and Biotechnologies, Sapienza University of Rome, 04100 Latina, Italy; Department of Radiological, Oncological and Pathological Sciences, Sapienza University of Rome, 00161 Rome, Italy; Unit of Endocrine Organs and Neuromuscolar Pathology, Fondazione Policlinico Universitario Campus Bio-Medico, 00128 Rome, Italy; Unit of Metabolic Bone and Thyroid Diseases, Fondazione Policlinico Universitario Campus Bio-Medico, 00128 Rome, Italy; Endocrinology, Diabetology and Metabolism, Department of Medical Sciences, University of Turin, 10126 Turin, Italy; Endocrinology, Diabetology and Metabolism, Department of Medical Sciences, University of Turin, 10126 Turin, Italy; Department of Medicine, Surgery and Neuroscience, Section of Endocrinology, University of Siena, 53100 Siena, Italy; Department of Medicine, Surgery and Neuroscience, Section of Endocrinology, University of Siena, 53100 Siena, Italy; Endocrine Oncology Unit, Department of Endocrine and Metabolic Diseases, IRCCS Istituto Auxologico Italiano, 20095 Milan, Italy; Endocrine Oncology Unit, Department of Endocrine and Metabolic Diseases, IRCCS Istituto Auxologico Italiano, 20095 Milan, Italy; IRCCS Fondazione G. Pascale, National Cancer Institute, 80131 Naples, Italy; IRCCS Fondazione G. Pascale, National Cancer Institute, 80131 Naples, Italy; Endocrinology Unit, Department of Experimental and Clinical Biomedical Sciences “Mario Serio”, University of Florence, 50134 Florence, Italy; Endocrinology Unit, Department of Experimental and Clinical Biomedical Sciences “Mario Serio”, University of Florence, 50134 Florence, Italy; Department of Medicine and Aging Sciences, University “G. d'Annunzio” of Chieti-Pescara, 66100 Chieti, Italy; Department of Medicine and Aging Sciences, University “G. d'Annunzio” of Chieti-Pescara, 66100 Chieti, Italy; Department of Clinical and Experimental Medicine, University of Catania, 95122 Catania, Italy; Department of Medical Sciences, Hospital “Casa Sollievo della Sofferenza”, IRCCS, 71013 San Giovanni Rotondo, Italy; Nuclear Medicine, Department of Medical and Surgical Specialties, Radiological Sciences, and Public Health, University of Brescia and Spedali Civili di Brescia, 25123 Brescia, Italy; UO Endocrinologia, Diabetologia e Malattie del metabolismo, AO Ordine Mauriziano Torino, 10128 Torino, Italy; Department of Surgery, Medical and Molecular Pathology and Critical Area, University of Pisa, 56100 Pisa, Italy; Operative Unit of Endocrinology Department of Medicine-DIMED University of Padua, 35128 Padua, Italy; Endocrine Unit, Tinchi Hospital, Asm Matera, 75015 Matera, Italy; Endocrinology and Diabetes Unit, Azienda Ospedaliero-Universitaria Sant’Andrea, Sapienza University of Rome, 00189 Rome, Italy; Department of Public Health, University of Naples “Federico II”, 80131 Naples, Italy; Unit of Endocrinology, Department of Biomedical, Metabolic and Neural Sciences, University of Modena and Reggio Emilia, 41125 Modena, Italy; Unit of Endocrinology, Department of Medical Specialties, Azienda Ospedaliero-Universitaria of Modena, 41124 Modena, Italy; Unit of Endocrinology ASL North West Tuscany, Ospedali Riuniti, 57124 Livorno, Italy; Department of Statistical Sciences, Sapienza University of Rome, 00185 Rome, Italy; Department of Statistical Sciences, Sapienza University of Rome, 00185 Rome, Italy; Department of Translational and Precision Medicine, Sapienza University of Rome, 00161 Rome, Italy; Department of Translational and Precision Medicine, Sapienza University of Rome, 00161 Rome, Italy

**Keywords:** thyroglobulin, threshold, predictor, treatment response, radioiodine

## Abstract

**Context:**

The utility of thyroglobulin (Tg) in the follow-up of patients with differentiated thyroid cancer has been well-documented. Although third-generation immunoassays have improved accuracy, limitations persist (interfering anti-Tg antibodies and measurement variability). Evolving treatment strategies require a reevaluation of Tg thresholds for optimal patient management.

**Objective:**

To assess the performance of serum Tg testing in 2 populations: patients receiving total thyroidectomy and radioiodine remnant ablation (RRA) or treated with thyroidectomy alone.

**Design:**

Prospective observational study.

**Setting:**

Centers contributing to the Italian Thyroid Cancer Observatory database.

**Patients:**

We included 540 patients with 5 years of follow-up and negative anti-Tg antibodies.

**Interventions:**

Serum Tg levels assessed at 1-year follow-up visit.

**Main Outcome Measure:**

Detection of structural disease within 5 years of follow-up.

**Results:**

After excluding 26 patients with structural disease detected at any time point, the median Tg did not differ between patients treated with or without radioiodine. Data-driven Tg thresholds were established based on the 97th percentile of Tg levels in disease-free individuals: 1.97 ng/mL for patients undergoing thyroidectomy alone (lower than proposed by the Memorial Sloan Kettering Cancer Center protocol and ESMO Guidelines, yet demonstrating good predictive ability, with a negative predictive value of 98% and 0.84 ng/mL for patients receiving postsurgical RRA. High sensitivity and negative predictive value supported the potential of these thresholds in excluding structural disease.

**Conclusion:**

This real-world study provides evidence for the continued reliability of 1-year serum Tg levels. The data-driven Tg thresholds proposed offer valuable insights for clinical decision-making in patients undergoing total thyroidectomy with or without RRA.

Thyroglobulin (Tg) serves as a pivotal marker in the management of patients with differentiated thyroid cancer (DTC) (1) because of its sensitivity and specificity for detecting residual or recurrent disease, facilitating treatment monitoring (2, 3). The utility of Tg in early and long-term follow-up of patients with DTC has been well-documented. For instance, Spencer et al (4). found that undetectable Tg levels after initial treatment correlated with a lower risk of recurrence, whereas increasing Tg levels suggested the presence of residual or recurrent disease. The availability of highly sensitive assays, such as third-generation Tg immunoassays, has significantly improved the accuracy of Tg measurements. These assays have enabled the detection of very low levels in patients with minimal disease burden, thereby enhancing the clinical utility of Tg as a tumor marker. However, Tg measurements have known limitations. They are unreliable in the presence of antithyroglobulin antibodies (5, 6), and there can be inter-assay variability, which has been investigated by several studies (4, 7, 8). Furthermore, the deescalation of DTC treatment shifted many patients from the systematic combination of total thyroidectomy and radioiodine remnant ablation (RRA) toward a more selective use of the latter procedure (9). The persistence of thyroid remnants may be a confounder of Tg results making the interpretation of low, detectable Tg levels more difficult. Finally, the thresholds proposed by the clinical practice guidelines (2, 3) are substantially based on expert opinion and data from retrospective studies of relatively small cohorts (10-13) and require validation studies.

This study aims to address these issues by examining a large, contemporary, prospectively enrolled cohort of patients with DTC. We sought to verify the difference in serum Tg levels after total thyroidectomy with or without thyroid remnant ablation during early follow-up. Additionally, we aimed to identify a data-driven Tg threshold (measured approximately 1 year after the initial treatment) and test its performance in the first 5 years of follow-up.

## Patients and Methods

The Italian Thyroid Cancer Observatory (ITCO), a web-based database, was started in 2013 at the Thyroid Cancer Center of Sapienza University of Rome, which serves as the network's coordinating center (14). The dataset includes prospectively collected data from more than 12 000 patients with histologically confirmed diagnoses of differentiated, poorly differentiated, anaplastic and medullary thyroid cancer from 51 participating thyroid cancer centers. The registered prospective study (ClinicalTrials.gov NCT04031339) has received ethical approval by the Coordinating Center Ethics Committee (Sapienza University of Rome, ref. 3366), as well as from the individual ethics committees associated with each participating center. Cases are included in the database at the time of initial treatment by the reporting ITCO center, or when the patient begins follow-up in the reporting center within 12 months after undergoing initial treatment in a non-ITCO center, after signing the informed consent form. We have already reported assessments of the baseline risk estimates on the first patients reaching early follow-up evaluations (15, 16) and an analysis of the role of extrathyroidal extension (17) and autoimmune thyroiditis (18). For this study, we reviewed all records available in the ITCO database and selected consecutive cases that satisfied the following criteria: (1) histological diagnosis of DTC; (2) initial treatment including total thyroidectomy (included patients who underwent completion thyroidectomy following thyroid lobectomy) with or without subsequent radioiodine treatment; (3) low or intermediate risk of recurrence according 2009 American Thyroid Association (ATA) guidelines (19) and relevant 2015 updates (2), based on data available immediately after the initial treatment; (4) availability of the results at 1-, 3-, and 5-year follow-up visits (after initial treatment ± 6 months), including all data required to classify the estimated treatment response; and (5) absence of anti-Tg antibody (TgAb) positivity. When treatment involved lobectomy followed by completion thyroidectomy, we considered histopathology data collected during both surgical procedures. The evaluation of the response to initial therapy was based on the collected clinical data, including imaging findings (cervical ultrasound for all patients and radioiodine scintigraphy for selected individuals) and basal (on levothyroxine) serum Tg and TgAb levels. Additional imaging studies were performed at the clinicians’ discretion. The results were classified as per the ATA guidelines for patients who underwent thyroidectomy followed by RRA, and as recommended by the European Society for Medical Oncology (3) for those whose initial treatment consisted of surgery alone. Patients were excluded if their TSH levels exceeded the third tertile of the distribution of the whole cohort at any assessment time.

### Tg Assays

Each participating center was required to provide the assay used and the corresponding limit of quantification (LOQ). According to the Clinical & Laboratory Standards Institute guidelines and the recent expert consensus (20), all assays used are highly-sensitive Tg assays (namely, Abbott Alinity i Tg, Abbott Architect Tg, Beckman Coulter Access Tg, BRAHMS Thermofisher h-Tg Sensitive KRYPTOR, Diasorin Liaison Tg II Gen, Roche Elecsys Tg II). In a small number of cases, a conventional test was adopted (Siemens Healthineers Immulite 2000 Tg or Diasorin Liaison Tg I gen). In all cases, values below the reported LOQ were considered equal to the LOQ for the analysis.

### Statistical Analysis

We summarized the distribution of continuous variables using medians and interquartile ranges, while nominal variables were described in terms of frequency counts and corresponding percentages. We used the chi-squared test or the Fisher exact test to assess significant association in contingency tables. The difference in mean of continuous variables across groups was evaluated using the Welch *t*-test. Our predictor, the serum unstimulated Tg level at 1 year after the initial treatment, was summarized using several measures: median, mean, interquartile range, range (min-max) and 97th percentile.

A new cutoff to discriminate the presence of structural disease was defined based on the 97th percentile of the putatively structural disease-free population. To validate the diagnostic accuracy of the new cutoff, we compared the expected information with the observed data. We calculated sensitivity, specificity, and positive and negative predictive values to verify the diagnostic performance of 1-year prediction. Confidence intervals are constructed for all previously specified measures.

To evaluate the difference in the central tendency of a continuous variable, that it is not normally distributed between groups, the Brown-Mood median test was used. The null hypothesis verifies that the medians of the population from which the groups were sampled are equals. All statistical analyses were performed using the R statistical software package (21).

## Results

**Figure 1.**
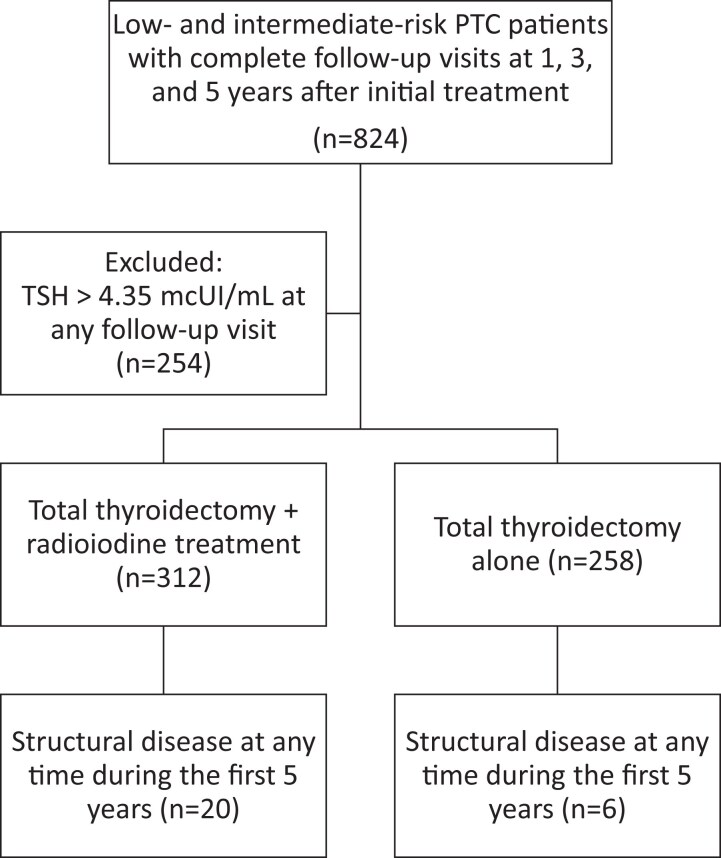
Study population.

A total of 824 patients fulfilled the inclusion criteria at the time of data analysis (2023). Serum TSH levels were evaluated, and tertiles were derived as follows: <0.1 mcUI/mL, 0.1-0.54 mcUI/mL, and 0.54-4.35 mcUI/mL. Subsequently, 254 patients were excluded because of TSH levels exceeding the upper normal level (above the upper value of the third tertile: 4.35 mcUI/mL) at any evaluation point, resulting in the final cohort of 540 patients (Fig. 1). The main clinical and demographic data, with the differences between the groups identified according to the 2 treatment modalities, total thyroidectomy with or without RRA, are presented in Table 1. We assessed the distribution of serum Tg at “1-year visit” (between 6 and 18 months after initial treatment), stratified by treatment choice.

**Table 1.** Clinical and demographic characteristics of the study population

		Total		TT + RRA		TT		*P^a^*
n		570		312		258		
Age (y)		49	40-59	48	38-57.3	50	41-60	.016
Sex	Male	151	26.5%	80	25.6%	71	27.5%	.7
	Female	418	73.5%	232	74.4%	186	72.1%	
Neck dissection	Central and lateral	55	9.7%	44	14.1%	11	4.3%	<.0001
	Central	174	30.5%	119	38.1%	55	21.3%	
	Lateral	12	2.1%	10	3.2%	2	0.8%	
	No formal neck dissection	329	57.7%	139	44.5%	190	73.6%	
PTC subtypes	Unknown	31	5.4%	15	4.8%	16	6.2%	.048
	Classic	276	48.4%	151	48.4%	125	48.4%	
	Follicular	209	36.7%	114	36.5%	95	36.8%	
	Tall cell	11	1.9%	10	3.2%	1	0.4%	
	Other	33	5.8%	14	4.5%	19	7.4%	
	Aggressive variants*^b^*	10	1.8%	8	2.6%	2	0.8%	
Tumor size (mm)		10	6-16	12	8-20.8	7	4-10	<.0001
Tumoral foci	Multifocal, bilateral	148	26.0%	103	33.0%	45	17.4%	<.0001
	Multifocal, not specified	4	0.7%	3	1.0%	1	0.4%	
	Multifocal, unilateral	69	12.1%	45	14.4%	24	9.3%	
	Not specified	3	0.5%	2	0.6%	1	0.4%	
	Unifocal	346	60.7%	159	51.0%	187	72.5%	
Extrathyroidal extension	No	395	69.3%	174	55.8%	221	85.7%	<.0001
	Microscopic	175	30.7%	138	44.2%	37	14.3%	
Lymph node metastases	N0	444	77.9%	204	65.4%	240	93.0%	<.0001
	N1a	70	12.3%	60	19.2%	10	3.9%	
	N1b	56	9.8%	48	15.4%	8	3.1%	
Vascular invasion	No	410	71.9%	204	65.4%	206	79.8%	<.0001
	Yes	56	9.8%	54	17.3%	2	0.8%	
	Unknown	104	18.2%	54	17.3%	50	19.4%	
ATA risk	Low	320	56.1%	111	35.6%	209	80.0%	<.0001
	Intermediate	250	43.9%	201	64.4%	49	19.0%	
1-y serum TSH	mcUI/mL (median, IQR)	0.20 (0.06-0.75)		0,11 (0.04-0.40)		0.5 (0.13-1.37)		<.0001
1-y response to initial treatment	SIR	19	3.33%	14	4.5%	5	1.90%	.13
	BIR	12	2.1%	9	2.9%	3	1.20%	
	IND	206	36.1%	115	38.9%	91	35.30%	
	ER	333	58.4%	174	55.8%	159	61.63%	
3-y serum TSH	mcUI/mL (median, IQR)	0.48 (0.16-1.17)		0.36 (0.13-1.0)		0.68 (0.26-1.39)		.0004
3-y response to initial treatment	SIR	12	2.1%	10	3.2%	2	0.80%	<.0001
	BIR	11	3.8%	9	2.9%	2	0.80%	
	IND	149	23.8%	55	17.6%	94	36.40%	
	ER	398	70.3%	238	76.30%	160	62.00%	
5-y serum TSH	mcUI/mL (median, IQR)	0.80 (0.30-1.72)		0.74 (0.25-1.58)		0.91 (0.4-1.96)		.03
5-y response to initial treatment	SIR	15	2.6%	13	4.20%	2	0.80%	<.0001
	BIR	8	1.4%	6	1.90%	2	0.80%	
	IND	144	25.3%	52	16.70%	92	35.70%	
	ER	403	70.7%	241	77.20%	162	62.80%	

Abbreviations: ATA, American Thyroid Association; BIR, biochemical incomplete response; ER, excellent response; IND, indeterminate response; PTC, papillary thyroid carcinoma; RRA, radioiodine ablation; SIR, structural incomplete response; TT, total thyroidectomy.

^*a*^Fisher test for categorical variables, Brown-Mood median test for continuous variables.

^*b*^Aggressive variants include solid, insular, tall cell, columnar cell, hobnail cell, and sclerosing, histologic subtypes.

As expected, the degree of TSH suppression changed over time and was modulated according to the initial treatment (Table 1) and baseline risk (Table 2). Therefore, we first examined the median, mean, interquartile range, and 97th percentile of serum Tg levels according to the corresponding serum TSH level. As expected, the distribution of Tg changed according to the TSH class, broadening with higher TSH values (0.54-4.3 mcUI/mL, within the median and the third tertile of the TSH distribution). Notably, within each TSH class, the median Tg did not differ between patients treated with or without RRA (Table 3).

**Table 2.** Response to treatment according to the initial estimate of the ATA risk

		Total		TT + RRA		TT		*P^a^*
Low risk of persistent or recurrent disease								
n		320		111		209		—
1-y TSH	mcUI/mL (median, IQR)	0.26 (0.08-0.95)		0.11 (0.05-0.36)		0.50 (0.13-1.32)		<.0001
1-y response to initial treatment	SIR	6	1.9%	3	2.7%	3	1.4%	.63
	BIR	8	2.5%	4	3.6%	4	1.9%	
	IND	114	35.6%	39	35.1%	75	35.9%	
	ER	192	60%	65	58.6%	127	60.8%	
3-y TSH	mcUI/mL (median, IQR)	0.53 (0.2-1.22)		0.36 (0.1-1.08)		0.67 (0.28-1.37)		.007
3-y response to initial treatment	SIR	2	0.6%	1	0.9%	1	0.5%	.005
	BIR	10	3.1%	3	2.7%	7	3.3%	
	IND	90	28.1%	19	17.1%	71	34%	
	ER	218	68.1%	88	79.3%	130	62.2%	
5-y TSH	mcUI/mL (median, IQR)	0.9 (0.35-1.73)		0.78 (0.3-1.54)		0.91 (0.41-1.94)		.29
5-y response to initial treatment	SIR	1	0.3%	1	0.9%	0	0%	.002
	BIR	10	3.1%	2	1.8%	8	3.8%	
	IND	84	26.3%	18	16.2%	66	31.6%	
	ER	225	70.3%	90	81.1%	135	64.6%	
Intermediate risk of persistent or recurrent disease								
n		250		201		49		—
1-y TSH	mcUI/mL (median, IQR)	0.13 (0.04-0.55)		0.11 (0.04-0.4)		0.8 (0.13-1.38)		<.001
1-y response to initial treatment	SIR	13	5.2%	11	5.5%	2	4.1%	.57
	BIR	16	6.4%	13	6.5%	3	6.1%	
	IND	80	32.0%	68	33.8%	12	24.5%	
	ER	141	56.4%	109	54.2%	32	65.3%	
3-y TSH	mcUI/mL (median, IQR)	0.4 (0.15-1.04)		0.38 (0.14-0.9)		0.72 (0.25-1.49)		.24
3-y response to initial treatment	SIR	10	4.0%	9	4.5%	1	2.0%	.03
	BIR	11	4.4%	10	5.0%	1	2.0%	
	IND	49	19.6%	32	15.9%	17	34.7%	
	ER	180	72.0%	150	74.6%	30	61.2%	
5-y TSH	mcUI/mL (median, IQR)	0.74 (0.25-1.71)		0.72 (0.22-1.6)		0.87 (0.34-2.06)		.26
5-y response to initial treatment	SIR	14	5.6%	12	6.0%	2	4.1%	.004
	BIR	10	4.0%	9	4.5%	1	2.0%	
	IND	49	19.6%	30	14.9%	19	38.8%	
	ER	177	70.8%	150	74.6%	27	55.1%	

Abbreviations: ATA, American Thyroid Association; BIR, biochemical incomplete response; ER, excellent response; IND, indeterminate response; RRA, radioiodine ablation; SIR, structural incomplete response; TT, total thyroidectomy.

^*a*^Fisher test for categorical variables, Brown-Mood median test for continuous variables.

**Table 3.** Median, mean, interquartile range, and 97th percentile of 1-y serum thyroglobulin values according to TSH tertiles and initial treatment

Treatment	TT + RRA					TT alone					
TSH	Median	Mean	IQR	Range	97th	Median	Mean	IQR	Range	97th	*P^a^*
<0.1	0.2	0.33	0.1-0.2	0-4.58	3	0.2	0.67	0.1-0.2	0-25	1.5	.43
0.1-0.54	0.2	0.3	0.1-0.2	0-7.9	1.16	0.2	0.98	0.1-0.31	0-30.7	4.5	.13
0.54-4.3	0.2	1.56	0.1-0.4	0-32.6	13.9	0.2	0.59	0.2-0.3	0-16	2.5	.21
	*P* value*^b^*						*P* value*^b^*				
1 vs 2	.98					1 vs 2	.05				
2 vs 3	.003					2 vs 3	.72				
1 vs 3	.001					1 vs 3	.01				

Abbreviations: IQR, interquartile range; RRA, radioiodine ablation; TT, total thyroidectomy.

^*a*^Comparison between serum Tg between different treatments for each TSH tertile.

^*b*^Comparisons between serum Tg between different TSH tertiles for each treatment group.

Our cohort included 26 patients with structural disease detected at any time point (Supplementary Table S1 (22)): these patients were managed according to the current practice guidelines. One patient had multiple neck lymph nodes confirmed by cytology, but no further surgery was performed because of the patient's age and comorbidities. In 4 cases, surgery was performed and confirmed metastatic lymph nodes. In 2 cases, radioiodine uptake was detected in the lung, and in 1 case in the mediastinum. In 14 cases there were small neck lymph nodes, with highly suspicious ultrasound features, managed conservatively (ie, no fine needle aspiration, no treatment), according to the ATA Guidelines’ recommendations. The remaining 4 cases were likely ultrasound false positive because they spontaneously disappeared over time.

**Figure 2.**
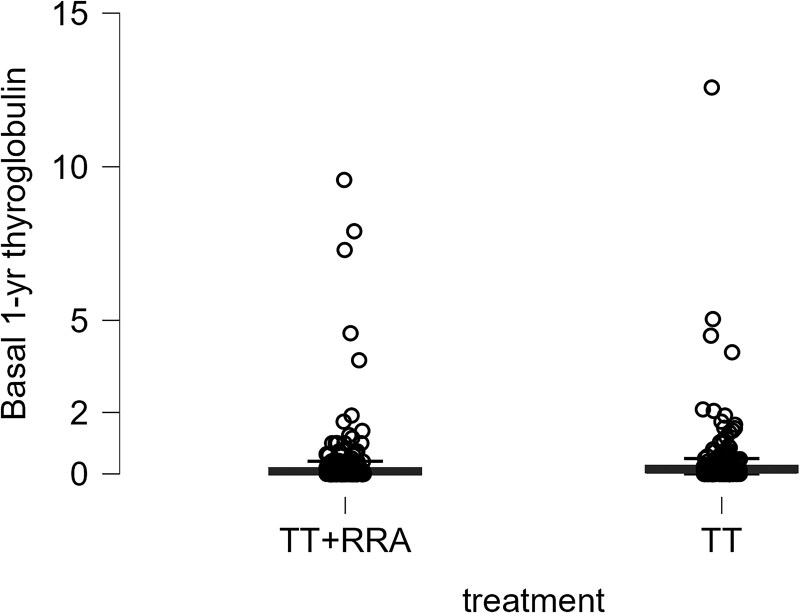
Distribution of 1-year serum thyroglobulin value in patients who underwent total thyroidectomy with or without radioiodine treatment.

After excluding these 26 patients with structural disease detected at any time point, the distribution of Tg levels remained unchanged between patients who underwent radioiodine treatment and those who did not (Fig. 2 and Table 4), as well as between patients with baseline low or intermediate risk of persistent or recurrent disease (Table 5). Given that the upper tails of the distributions appear to be more influenced by factors such as TSH stimulation (and potentially by the presence of residual thyroid tissue), we adopted the 97th percentile of patients with no structural evidence of disease as a potential cutoff. The 97th percentile of 1-year serum Tg levels was 0.84 ng/mL in patients undergoing RRA, and 1.97 ng/mL in patients undergoing thyroidectomy alone. We then evaluated the diagnostic performance of these thresholds as prognostic cutoffs in the entire cohort (Table 6). As expected, the sensitivity of serum Tg in patients treated with surgery alone is lower than in patients treated with RRA, but the negative predictive value of serum Tg to rule out structural disease is consistently higher than 95%. Notably, only 3 patients requiring further treatment had false-negative Tg (that is, below the threshold) values at 1-year evaluation (Supplementary Table S1 in the referenced repository) (22).

**Table 4.** 1-y serum thyroglobulin value distribution in patients undergoing total thyroidectomy with or without radioiodine treatment, showing no evidence of structural disease during the first 5 years of follow-up

1-y basal serum thyroglobulin		
Treatment	TT + RRA	TT alone
Patients	n = 292	n = 252
Median	0.1 ng/mL	0.11 ng/mL
Maximum	3.7 ng/mL	18.0 ng/mL
**97th percentile**	**0.84 ng/mL**	**1.97 ng/mL**

Abbreviations: RRA, radioiodine ablation; TT, total thyroidectomy.

**Table 5.** 1-y serum thyroglobulin value distribution in patients with low or intermediate risk of recurrence, showing no evidence of structural disease during the first 5 years of follow-up

1-y basal serum thyroglobulin		
ATA risk	Low	Intermediate
Patients	n = 314	n = 230
Median	0.1 ng/mL	0.1 ng/mL
Maximum	18 ng/mL	3.7 ng/mL
97th percentile	1.66 ng/mL	1.02 ng/mL

Abbreviation: ATA, American Thyroid Association.

**Table 6.** Diagnostic performance of 1-y serum thyroglobulin value in prediction of structural disease within 5 y (applying the newly defined cutoffs and the ESMO Guidelines cutoff)

	Sensitivity (95% CI)	Specificity (95% CI)	PPV (95% CI)	NPV (95% CI)
TT only	16.7% (0.4-64.1%)	96.8% (93.8-98.6%)	11.1% (10.3-48.2%)	98% (95.4-99.4%)
TT + RRA	40% (19.1-63.9%)	96.9% (94.2-98.6%)	47.1% (23-72.2%)	95.9% (93-97.9%)
ESMO Guidelines cutoff				
TT only	16.7% (0.4-64.1%)	98.4% (96-99.6%)	20% (0.5-71.6%)	98% (95.4-99.4%)
TT + RRA	40% (19.1-63.9%)	96.9% (94.2-98.6%)	47.1% (23-72.2%)	95.9% (93-97.9%)

Abbreviations: NPV, negative predictive value; PPV, positive predictive value; RRA, radioiodine ablation; TT, total thyroidectomy.

## Discussion

The Tg thresholds proposed by guidelines for various clinical scenarios in thyroid cancer follow-up are derived from a combination of expert consensus and retrospective studies. Specifically, the referenced studies evaluated the diagnostic accuracy, prognostic value, and clinical utility of Tg measurements with the threshold suggested at the Memorial Sloan Kettering Cancer Center in New York, and subsequently validated (10, 12, 13). Expert panels then considered these findings alongside their clinical experience and judgment to formulate guideline recommendations.

However, the clinical landscape for papillary thyroid cancer has shifted significantly in recent years. We are witnessing a rise in diagnosis of small papillary tumors, with a corresponding trend toward less aggressive treatment approaches. Notably, the use of RRA treatment as a complement to surgery has seen a dramatic decline. This study aims to assess the validity of Tg testing in this contemporary setting, focusing on 2 patient populations: those receiving traditional therapy (total thyroidectomy and RRA) and those treated solely with thyroidectomy. By evaluating Tg levels in these groups, we hope to establish more relevant reference ranges and validate its continued use as a clinical marker.

A recent systematic review examined the diagnostic accuracy of serum Tg measurement for structural disease in patients treated with total or near-total thyroidectomy, without radioiodine (23). Five studies evaluated Tg measurement in this context (11, 24-27). However, the timing of initial Tg measurement varied across studies, and 2 studies lacked this information (11, 27). Additionally, TSH status was reported only in 1 study (24), whereas TgAb status was inconsistently reported, with 1 study failing to report it (27). Thus, the review advocated for additional research to clarify the role of Tg measurement in these settings and determine optimal thresholds (23). In this regard, our study sought to determine the best Tg thresholds at precise timepoints while accounting for TSH values and excluding all cases with positive TgAbs.

Our data confirm that 1-year serum Tg level is a usable biomarker in patients with differentiated thyroid cancer. We have detected 2 potential data-derived threshold (97th percentile of apparently structural disease-free individuals): 0.84 ng/mL in patients submitted to radioiodine treatment (comparable to the 1 ng/mL cutoff proposed by the current guidelines), and 1.97 ng/mL in patients submitted to thyroidectomy alone (lower than proposed by the Memorial Sloan Kettering Cancer Center protocol and by the ESMO Guidelines, but with high negative predictive value 98%). In patients with minimal residual structural disease, at least at an early stage of detection, Tg may be falsely negative (Supplementary Table S1 (22)). This justifies the concomitant use of first-level imaging techniques in the initial follow-up period to further increase the positive predictive value.

As with any real-world studies, this study has several limitations. First, 8 different Tg immunoassays were used in the participating clinical centers, with some changes over time. Regrettably, despite calibration against an international reference standard (BRC 457), differences are documented across assays (even in studies in which multiple assays were used to analyze the same samples) because of the heterogeneity in Tg structure and assay technical specifications. Although the use of 8 different Tg assays across centers introduces variability, it also reflects the real-world scenario where diverse assays are routinely employed. The cutoffs derived in this study, therefore, offer a reference point relevant to what is commonly observed in clinical practice. However, given the critical role of Tg assays in DTC follow-up, improved standardization and control remain highly desirable (20). Furthermore, we had a small rate of structural disease detection because of the selection of cases with baseline low to intermediate risk. The proposed thresholds need to be further validated in independent and external cohorts. Finally, we had to exclude all cases with positive TgAb, according to the evaluation of the treating clinicians.

Despite all these limitations, our findings offer a contemporary reference point for the 1-year unstimulated serum Tg cutoff values in a clinical landscape that is characterized by an increased detection of small cancers, a decreased frequency of total thyroidectomy and a reduction in the use of RRA. Given its accessibility and cost-effectiveness, Tg remains the primary marker for monitoring the majority of patients in this new clinical context.

## Disclosures

None of the authors has a conflict of interest that is relevant to the subject matter or materials included in this work. G.G. is an editorial board member for *The Journal of Clinical Endocrinology & Metabolism* and played no role in the Journal's evaluation of the manuscript.

## Data Availability

Restrictions apply to the availability of some or all data generated or analyzed during this study to preserve patient confidentiality or because they were used under license. The corresponding author will on request detail the restrictions and any conditions under which access to some data may be provided.

## Abbreviations

ATA: American Thyroid Association
DTC: differentiated thyroid cancer
ITCO: Italian Thyroid Cancer Observatory
LOQ: limit of quantification
RRA: radioiodine remnant ablation
Tg: thyroglobulin
TgAb: antithyroglobulin antibody
